# A Comparison of Diagnosed Skin Diseases between the Years with and without COVID-19 Pandemic

**DOI:** 10.3390/medicina57080773

**Published:** 2021-07-29

**Authors:** Wanjarus Roongpisuthipong, Pornchai Yodla, Theerawut Klangjareonchai

**Affiliations:** 1 Department of Medicine, Division of Dermatology, Faculty of Medicine Vajira Hospital, Navamindradhiraj University, Bangkok 10300, Thailand; 2 Department of Medical Records and Statistics, Faculty of Medicine Vajira Hospital, Navamindradhiraj University, Bangkok 10300, Thailand; Pornchai@nmu.ac.th; 3 Department of Medicine, Faculty of Medicine, Ramathibodi Hospital, Mahidol University, Bangkok 10400, Thailand; theerawutklang@gmail.com

**Keywords:** coronavirus disease 2019 (COVID-19) pandemic, skin diseases, outpatient dermatology clinic, dermatitis, cutaneous infection, urbanology

## Abstract

*Background and Objectives*: The COVID-19 pandemic has a considerable influence on public health, either directly or indirectly. We investigated outpatient skin disease diagnoses at the dermatology clinic to determine the effect of the COVID-19 pandemic on these patients. *Materials and Methods*: We conducted a retrospective study using the International Codes of Diseases data from the outpatient department of Dermatology clinic, Vajira hospital, Navamindradhiraj University, Bangkok, Thailand from January 2019 to June 2021. *Results*: A total of 20,915 patients with 34,116 skin diagnoses were included in the study. The average weekly dermatologic clinic visits remained unchanged between the years with and without COVID-19 pandemic. While the percentage of xerosis cutis, other skin infections (syphilis and parasitic infections), hair and nail disorders, pigmentary disorders, benign skin tumors and drug eruptions were significantly decreased during the COVID-19 pandemic years, the percentage of other dermatitis, fungal and viral skin infections, acne, psoriasis, urticaria, vesiculobullous and autoimmune diseases were increased. *Conclusion*: The COVID-19 pandemic had a minimal effect on the average weekly skin clinic visits, but the diagnosed skin diseases pattern was affected. Knowing the pattern of skin diseases may help aid hospitals to better prepare for future pandemics in securing appropriate medications and supplies and training the medical teams.

## 1. Introduction

The novel coronavirus disease 2019 (COVID-19) outbreak, which is caused by the severe acute respiratory syndrome coronavirus 2 (SARS-CoV-2) virus strain, has been spreading around the world since late December 2019 [1]. The virulent viral pneumonia outbreak in the city of Wuhan, Hubei province, China and has affected people all over the world. The first case of COVID-19 in Thailand was reported on 12 January 2020, after a 61-year-old Chinese woman, from Wuhan, China, had visited Thailand. At the beginning of the pandemic, the majority of the COVID-19 cases were related to foreign visitors, taxi drivers, bus drivers and shopping mall employees. The number of cases steadily increased and clusters of cases emerged at Thai boxing events and nightlife establishments throughout Bangkok. The first case of COVID-19 death was reported in March 2020. This prompted the Thai government to enforce the Emergency Decree on Public Administration in Emergency Situations from 26 March to 30 April 2020 to curb the COVID-19 outbreak [2,3]. People adapted new daily lifestyles such as working from home, shopping online, attending virtual meetings and studying online. To prevent the spread of COVID-19, healthy hygiene habits are recommended, such as wearing a face mask, washing hands often and thoroughly with soap or alcohol-based hand sanitizer, and keeping a safe physical distance to protect the transmission [4,5]. Although people avoided going to hospitals during this pandemic because of the fear of COVID-19 infection, some patients with dermatologic diseases still need to visit dermatologists. This is an extraordinarily challenging time for all medical staff, including dermatologists [6]. Knowing the pattern of skin diseases may help aid hospital to better prepare for future pandemic in securing appropriate medications and supplies and training the medical teams. This study aims to compare the encountered diagnosed skin diseases at the clinic to determine the effect of the COVID-19 pandemic on these patients.

## 2. Materials and Methods

### 2.1. Study Design, Setting and Participants

This study was carried out as a retrospective study by assessing the numbers and skin diagnoses of patients who visited the outpatient department (OPD) of the dermatology clinic at Vajira Hospital, Navamindradhiraj University, Bangkok, Thailand during January 2019 and June 2021. The board-certified dermatologists are responsible for diagnosing and managing all patients. The electronic medical databases of these patients were reviewed.

For this study, the COVID-19 pandemic time period is defined from January 2020 to June 2021 to reflect the series of outbreaks in Thailand. The first case of COVID-19 was reported in Thailand on 12 January 2020. The first wave of the COVID-19 outbreak occurred between March 2020 and May 2020. The second wave of the disease started in late December 2020 and the third wave of the COVID-19 outbreak was in April 2021. Inclusion criteria included patients over the age of 15 with skin problems who visited the dermatology clinic between January 2019 and June 2021. Exclusion criteria were patients who were admitted to the hospital with incomplete data records and non-skin complaints. The International Classification of Diseases, tenth revision (ICD-10) codes were used to identify diagnosed skin diseases in patients. ICD-10 codes were classified into fifteen different skin disease categories, including dermatitis (atopic dermatitis, contact dermatitis, seborrheic dermatitis, and other dermatitis), cutaneous infection (fungal diseases, viral diseases, bacterial diseases, and other infections), sebaceous gland disorders (acne vulgaris and rosacea), psoriasis, xerosis cutis, urticaria, pigmentary disorders (post inflammatory hyper/hypopigmentation, vitiligo, chloasma, other pigmentation disorders), hair and nail disorders, drug allergy, cutaneous neoplasms (benign and malignancy), vesiculobullous diseases (bullous pemphigoid, pemphigus vulgaris, pemphigus foliaceus, other vesiculobullous diseases), autoimmune diseases (Lupus erythematosus, systemic sclerosis, dermatomyositis, other autoimmune disorders), vascular disorders (vascular malformation, vasculitis, other vascular diseases), papulosquamous disorders (lichen planus, pityriasis lichenoides chronica, mycosis fungoides, other papulosquamous diseases), and other skin diseases.

### 2.2. Statistical Analysis

The main result was a comparison of the distribution of diagnosed skin diseases at the dermatologic outpatient unit between January 2019 and June 2021. For continuous data, the mean and standard deviation were used to describe normally distributed data and were compared using the independent *t*-test. Categorical data was presented with frequency and percentage. For categorical data comparison, the Chi-square test was used. All statistical data analysis was carried out using Stata version 13 (Stata, College Station, TX, USA). A *p*-value of 0.05 or less was defined as statistically significant.

### 2.3. Ethical Aspects

This study was approved by the Institutional Review Board of the Faculty of Medicine Vajira Hospital, which waived the requirement of obtaining informed consent (Certificate of Approval: 025/2564). 

## 3. Results

### 3.1. Clinical Characteristics of Study Population

Data was gathered from 1 January 2019 to 30 June 2021; the total number of OPD visits was 20,915 with 34,116 diagnosed skin diseases. The year prior to the COVID-19 outbreak, 2019, revealed 8469 visits. The total number of patients treated during the years of the COVID-19 outbreak was 12,446. During the period of COVID-19 outbreak, the average weekly dermatologic clinic visits were 157.5 ± 39.1 compared to 162.9 ± 32.5 the previous year. The mean age of patients during the COVID-19 outbreak was 55.7 ± 18.1 years old, compared to 55.1 ± 18.3 years old in the comparative year. Female patients predominated in both periods, before and during COVID-19 outbreak. Of the 20,915 patients, 13,163 (63%) were female and 7752 (37%) were male. The majority of the patients lived in Bangkok, the capital of Thailand. The demographic data was shown in Table 1.

### 3.2. The Average Outpatient Dermatology Clinic Visits and Important COVID-19 Events in Thailand

**Figure 1:**
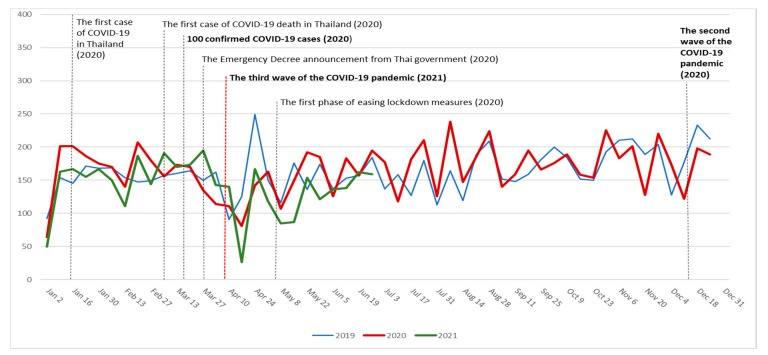
The number of dermatology clinic visits per week from 1 January 2019 to 30 June 2021. The dates of the major events are represented by the vertical lines. The blue line represents 2019, the red line represents 2020 and the green line represents 2021.

The number of clinic visits were impacted by the COVID-19 outbreaks. After the first report of COVID-19 death and 100 confirmed cases in 2020, the Thai government implemented the Emergency Decree on Public Administration in Emergency Situations. As people were directed to stay at home, the number of outpatient dermatology clinic visits declined. As the lockdown was lifted, the number of patient visits returned to normal and was maintained throughout the second wave in late December 2020. The number of patients visiting the OPD decreased after the third wave of COVID-19 outbreak in April 2021. The graphs compare the number of patients visiting dermatology OPD daily, as shown in Figure 1.

### 3.3. Distribution of Diagnosed Skin Diseases at Outpatient Dermatology Clinic

The five most common skin disease category rankings were the same in both periods between the years with and without the COVID-19 pandemic. Diagnosed skin diseases as ranking in the periods without and with COVID-19 pandemic are shown in order. The top skin disease category rank was dermatitis, 39.4% and 41.5%; skin infections, 13.5% and 14.1%; xerosis cutis, 9.3% and 8.5%; psoriasis, 7.8% and 8.5%; and cutaneous neoplasms, 4.5% and 3.6%. The periods without and with COVID-19 epidemic were shown in order, other dermatitis, 32.6% and 35%; fungal skin infections, 4.5% and 5.1%; viral skin infections, 6.0% and 6.6%; psoriasis, 7.8% and 8.5%; urticaria, 3.5% and 3.9%; acne, 2.3% and 3%; vesiculobullous diseases, 1.2% and 1.6%; and autoimmune diseases, 1.8 and 2.3%. These diagnosed skin diseases had a higher percentage of outpatient visits during the period of the COVID-19 epidemic. On the other hand, data of the compared periods without and with COVID-19 pandemic was shown, respectively; diagnosis of other skin infections, which include syphilis and parasitic infections, 1.0% and 0.6%; xerosis cutis, 9.3% and 8.5%; hair and nail disorders, 5.0% and 3.3%; benign skin tumors, 4.3% and 3.3%; pigmentary disorders, 2.7% and 2%; and drug eruptions, 0.5% and 0.3% had a lower percentage of outpatient visits in the year of the COVID-19 epidemic. Data was shown in detail in Table 2. 

## 4. Discussion

The number of patients visiting as outpatients of the dermatology unit decreased after the first episode of the COVID-19 outbreak in Thailand and continued to decline after the National Emergency Decree was announced. This finding was similar to the data reported at the skin clinic of a tertiary hospital in Turkey during a partial curfew. The number of patients who visited the skin clinic decreased from five to one [7]. After the lockdown, the number of daily dermatologic clinic visits eased back to normal. There was no significant difference in the total visits between the periods with and without the COVID-19 outbreak; this may be attributed to the effectiveness of Thailand’s government in controlling the disease transmission. On 6 January 2021, the total number of COVID-19 cases in Thailand was 9331 (134 cases per million population) with 66 deaths (0.9 death per million population) [8,9]. The number of patient visits declined in April 2021 after the third wave of COVID-19. As of 30 June 2021, Bangkok and its vicinity are the epicenter of this wave with 230,438 confirmed cases and 1929 deaths. This wave was uncontrolled, possibly due to the prevalence of more virulent strain and a delayed effort in COVID-19 vaccination. Approximately 2,762,537 people or 4% of population in Thailand are vaccinated. Moreover, the spread dynamics of COVID-19 depended upon the potential impact of stochasticity [10].

During the COVID-19 pandemic years, the percentage of xerosis cutis, other skin infections (syphilis and parasitic infections), hair and nail disorders, pigmentary disorders, benign skin tumors and drug eruptions decreased, according to the present study. On the contrary, this study found that the percentage of diseases such as other dermatitis, fungal and viral skin infections, acne, psoriasis, urticaria, vesiculobullous and autoimmune diseases had increased. The degree of anxiety and stress in humans rose around the world during the COVID-19 pandemic, including in Thailand [11]. The study from one of the communities in Bangkok found that there was an increase in stress levels during the COVID-19 pandemic which was associated with food insecurity and financial constraints [12]. Acne, psoriasis, urticaria and viral skin infections (warts, herpes simplex and herpes zoster) were all significantly increased. These skin diseases were characterized as psychophysiological disorders exacerbated by stress [13]. The increase in psoriasis, urticaria and herpes zoster had also been reported in studies form Turkey during the stressful pandemic [14,15]. Stress is one of many factors that can trigger vesiculobullous diseases such as pemphigus vulgaris, pemphigus foliaceus and bullous pemphigoid [16]. As a result of the negative emotional effect of the pandemic, vesiculobullous disease might be on the rise [17,18]. Furthermore, vesiculobullous patients typically used immunosuppressive drugs and need to visit hospital regularly despite the concerns regarding COVID-19 spreading. Patients with autoimmune diseases such as systemic lupus erythematosus, dermatomyositis and systemic sclerosis visiting the skin clinic in the pandemic period were higher than in the non-pandemic period. It is assumed that psychological stress causes immune dysregulation and amplified cytokine production, thereby exacerbating autoimmune diseases and decreasing host defense [19]. According to the study of cutaneous manifestation and SARS-CoV-2 infection, the findings of erythematous maculopapular, chilblain-like, urticaria, vesicular, livedo or necrosis and petechial lesions were shown in order. Awareness of these might explain the increase in the number of patients, such as urticaria and vesiculobullous diseases, in the COVID-19 pandemic period in this study [20,21].

There was a reduction in xerosis cutis during the COVID-19 pandemic years compared with the year without the COVID-19 pandemic. As people are staying at home and spending less time on outdoor activities, there are less chances of being exposed to pollutants and less showering is required. Ultraviolet radiation (UVR), air pollutants such as particulate matter (PM), polycyclic aromatic hydrocarbons (PAH) including ozone can increase dry skin [22]. The reduction of UVR and air pollution exposure might help epidermal barrier function as well as the synthesis of natural moisturizing factors [23,24]. Less frequent cleaning of the body, using soap without fragrance or syndet will always result in less destruction of the skin barrier [25]. Other dermatitis significantly increased in the years of the COVID-19 outbreak. Hand eczema was the main problem in this category. Dyshidrosis and hand eczema are diseases directly related to hand washing with or without soap or alcohol-based sanitizers. Frequent and thorough hand washing is known as one of the best ways to avoid SARS-CoV-2 virus infection [26]. However, it can inevitably result in a weakened skin barrier [27,28]. In addition, fungal skin infections might arise if the skin barrier structure is damaged [29]. This corresponded to an increase in the percentage of patients in this study who had fungal skin infections during the pandemic years. This study showed a significant increase in candida and dermatophyte infection. Chronic paronychia, implicated by candida infection, has a direct relation with frequent hand washing [30]. However, clinical nail changes of chronic paronychia such as onycholysis, nail plate dystrophy and candida onychomycosis might take time. This explains why nail disorders in the current study did not increase. Furthermore, the top of comorbid skin diseases of COVID-19 is superficial fungal skin infections. Patients with superficial fungal infections were more vulnerable to COVID-19 due to impaired cutaneous and mucosal immunity [31]. When compared to the previous year, the COVID-19 pandemic years showed that hair and nail issues were reduced by 29% and 42.1%, respectively. A multicenter study conducted in Turkey also found a reduction in hair and nail disorders in the COVID-19 epidemic [14]. Additionally, the number of pigmentary disorders and benign skin tumors declined significantly in this study. From the patients’ perspectives, the problems such as hairs and nails, pigmentary disorders and benign skin tumors might be cosmetic issues and the visit to the doctor could be postponed, especially during the pandemic. A faltering economy and limited discretionary expenditure also prevent people from undergoing costly aesthetic treatments [32]. From our data, malignant skin tumors, mainly non-melanoma skin cancer, remain unchanged. There was no difference in the average waiting time from diagnosis to surgery in pandemic and non-pandemic periods, which were 22 and 23 days, respectively. There was no increase in the severity of skin cancers in the COVID-19 pandemic period. This is in contrast to the findings from the study in Italy that showed an increase in advanced malignant skin tumors due to delaying the follow up, leading to postponed surgery [33]. Other skin infections, including parasitic skin infections, i.e., scabies, head lice, etc., were shown to be lower in the COVID-19 pandemic period, probably due to social distancing and effective hand hygiene, which reduces direct skin infection.

In this study, drug eruptions were significantly reduced in the COVID-19 pandemic period. Antibiotics are the most common culprit drug class [34]. In Thailand, antibiotics can be obtained over the counter without a doctor’s prescription. The majority of drugstore workers dispensed antibiotics for viral upper respiratory tract infections [35,36]. Measures to minimize viral transmission, such as washing hands, wearing facemasks and social distancing, were efficient for SARS-CoV-2 and any respiratory viral infections [37,38]. This might explain the declining usage of inappropriate oral antibiotics in Thailand.

The strengths of this study include the large sample size, two-and-a-half-year study period and all diagnosed diseases made by board-certified dermatologists. The limitations of this study include that all the data were from tertiary care centers in urban areas. The results are suitable to adapt with the same level of healthcare settings but do not represent rural areas. In addition, because this study is a retrospective study, other factors influencing the number of clinic visits at the dermatology outpatient center could not be determined. In order to better understand the real situation, further studies evaluating the pattern of diagnosed skin diseases in multicenter settings, including both urban and rural areas, are essential.

## 5. Conclusions

This study compares the number of skin diseases diagnosed in an outpatient service in a tertiary care hospital during the COVID-19 pandemic years to the prior year. The period of COVID-19 outbreak had a minimal effect on average daily skin clinic visits, but the pattern of skin diseases changed. In the event of a subsequent outbreak, providing data could help hospitals to deal with dermatological patient’s management, as well as educational initiatives and preventive strategies.

## Figures and Tables

**Table 1:** A comparison of demographic characteristics of patients visiting outpatient dermatology clinic at Vajira Hospital, Navamindradhiraj University, between the years with and without COVID-19 outbreak.

	Before COVID-19 Outbreak Period (*n* = 8469)	During COVID-19 Outbreak Period (*n* = 12,446)	*p* Value
Age (years) *	55.1 ± 18.3	55.7 ± 18.1	0.025
Gender, *n* (%)			
Male	3109 (36.7)	4643 (37.3)	0.382
Female	5360 (63.3)	7803 (62.7)	
Residence, *n* (%)			
Bangkok	6069 (71.7)	8942 (71.8)	0.77
Others	2400 (28.3)	3504 (28.2)	
Visits per week	162.9 ± 32.5	157.5 ± 39.1	0.417

Data are shown as the mean ± SD. * *p* < 0.05 significantly different by independent *t*-test and Chi-square test.

**Table 2:** A comparison of distribution of diagnosed skin diseases among patients visiting outpatient dermatology clinic at Vajira Hospital, Navamindradhiraj University, between the periods with and without COVID-19 outbreak.

	Before COVID-19 Outbreak Period (*n* = 13,820)		During COVID-19 Outbreak Period (*n* = 20,296)		Period Difference	*p* Value
Categories	*n*	%	*n*	%	%	
Dermatitis						
Contact dermatitis	358	2.6	552	2.7	3.8	0.209
Seborrheic dermatitis	511	3.7	697	3.4	−8.1	0.226
Atopic dermatitis	69	0.5	78	0.4	−20.0	0.081
Other dermatitis *	4503	32.6	7098	35	7.4	<0.001
Cutaneous infections						
Viral diseases *	829	6.0	1342	6.6	10.0	<0.001
Fungal diseases *	620	4.5	1043	5.1	13.3	<0.001
Bacterial diseases	276	2.0	371	1.8	−10.0	0.217
Other infections *	140	1.0	112	0.6	−40.0	<0.001
Psoriasis *	1079	7.8	1717	8.5	9.0	<0.001
Xerosis cutis *	1280	9.3	1715	8.5	−8.6	<0.001
Urticaria *	477	3.5	799	3.9	11.4	<0.001
Cutaneous neoplasms						
Benign *	596	4.3	678	3.3	−23.3	<0.001
Malignancy	23	0.2	55	0.3	50.0	0.226
Sebaceous gland disorders						
Acne *	315	2.3	603	3.0	30.4	<0.001
Rosacea	20	0.1	28	0.1	0.0	0.805
Hair disorders *	431	3.1	437	2.2	−29.0	<0.001
Nail disorders *	269	1.9	228	1.1	−42.1	<0.001
Pigmentary disorders *	371	2.7	400	2.0	−29.0	<0.001
Vesiculobullous diseases *	170	1.2	332	1.6	33.3	0.022
Autoimmune diseases *	249	1.8	469	2.3	27.8	<0.001
Vascular disorders	192	1.4	259	1.3	−7.1	0.298
Papulosquamous disorders	89	0.6	107	0.5	−16.7	0.115
Drug eruptions *	72	0.5	68	0.3	−40.0	0.005
Other skin diseases *	881	6.4	1108	5.5	−14.1	<0.001

* *p* < 0.05 significantly different by Chi-square test.

## Data Availability

The data presented in this study are available on request from the corresponding author.

## Abbreviations

COVID-19	coronavirus disease 2019
SARS-CoV-2	severe acute respiratory syndrome coronavirus 2
OPD	outpatient department
ICD-10	International Classification of Diseases, tenth revision
UVR	ultraviolet radiation
PM	particulate matter
PAH	polycyclic aromatic hydrocarbon

## References

[1] Li Q., Guan X., Wu P., Wang X., Zhou L., Tong Y., Ren R., Leung K.S., Lau E.H., Wong J.Y. (2020). Early transmission dynamics in Wuhan, China, of novel coronavirus–infected pneumonia. N. Eng. J. Med..

[2] Sriwijitalai W., Wiwanitkit V. (2020). Letter to the Editor: COVID-19 in a Thai Boxing Area in Thailand. Dtsch. Z. Sportmed..

[3] Malathum K., Malathum P. (2020). The COVID-19 Pandemic: What We Have Learned from Thai Experiences. Pac. Rim Int. J. Nurs. Res..

[4] Block R., Berg A., Lennon R.P., Miller E.L., Nunez-Smith M. (2020). African American adherence to COVID-19 public health recommendations. Health Lit. Res. Pract..

[5] Callaghan T., Lueck J.A., Trujillo K.L., Ferdinand A.O. (2021). Rural and urban differences in COVID-19 prevention behaviors. J. Rural Health.

[6] Wollina U. (2020). Challenges of COVID-19 pandemic for dermatology. Dermatol. Ther..

[7] Cengiz F.P., Emiroglu N., Bahali A.G., Dizman D., Taslidere N., Akarslan T.C., Gunes B., Mert O., Kucuk O.S., Onsun N. (2020). Which dermatology patients attend to Dermatology Outpatient Clinics during the SARS-CoV-2 outbreak in Turkey and what happened to them?. Dermatol. Ther..

[8] Marome W., Shaw R. (2021). COVID-19 response in Thailand and its implications on future preparedness. Int. J. Environ. Res. Public Health.

[9] Tangcharoensathien V., Bassett M.T., Meng Q., Mills A. (2021). Are overwhelmed health systems an inevitable consequence of covid-19? Experiences from China, Thailand, and New York State. BMJ.

[10] Fang Y., Nie Y., Penny M. (2020). Transmission dynamics of the COVID-19 outbreak and effectiveness of government interventions: A data-driven analysis. J. Med. Virol..

[11] Goodwin R., Wiwattanapantuwong J., Tuicomepee A., Suttiwan P., Watakakosol R., Ben-Ezra M. (2021). Anxiety, perceived control and pandemic behaviour in Thailand during COVID-19: Results from a national survey. J. Psychiatr. Res..

[12] Pongutta S., Kantamaturapoj K., Phakdeesettakun K., Phonsuk P. (2021). The social impact of the COVID-19 outbreak on urban slums and the response of civil society organisations: A case study in Bangkok, Thailand. Heliyon.

[13] Koo J., Lebwohl A. (2001). Psychodermatology: The mind and skin connection. Am. Fam. Physician.

[14] Kartal S.P., Çelik G., Sendur N., Aytekin S., Serdaroğlu S., Doğan B., Yazıcı A.C., Çiçek D., Borlu M., Kaçar N.G. (2020). Multicenter study evaluating the impact of COVID-19 outbreak on dermatology outpatients in Turkey. Dermatol. Ther..

[15] Kutlu Ö., Güneş R., Coerdt K., Metin A., Khachemoune A. (2020). The effect of the “stay-at-home” policy on requests for dermatology outpatient clinic visits after the COVID-19 outbreak. Dermatol. Ther..

[16] Tavakolpour S. (2018). Pemphigus trigger factors: Special focus on pemphigus vulgaris and pemphigus foliaceus. Arch. Dermatol. Res..

[17] Rania M., Petersen L.V., Benros M.E., Liu Z., Diaz L., Bulik C.M. (2020). Psychiatric comorbidity in individuals with bullous pemphigoid and all bullous disorders in the Danish national registers. BMC Psychiatry.

[18] Kluger N., Pankakoski A., Panelius J. (2020). Depression and Anxiety in Patients with Bullous Pemphigoid: Impact and Management Challenges. Clin. Cosmet. Investig. Dermatol..

[19] Stojanovich L., Marisavljevich D. (2008). Stress as a trigger of autoimmune disease. Autoimmun. Rev..

[20] Zhao Q., Fang X., Pang Z., Zhang B., Liu H., Zhang F. (2020). COVID-19 and cutaneous manifestations: A systemic review. J. Eur. Acad. Dermatol. Venereol..

[21] Suchonwanit P., Leerunyakul K., Kositkuljorn C. (2020). Diagnostic and prognostic values of cutaneous manifestations in COVID-19. Dermatol. Ther..

[22] Parrado C., Mercado-Saenz S., Perez-Davo A., Gilaberte Y., Gonzalez S., Juarranz A. (2019). Environmental stressors on skin aging. Mechanistic insights. Front. Pharmacol..

[23] Visser M.J., Landeck L., Campbell L.E., McLean W.H.I., Weidinger S., Calkoen F., John S.M., Kezic S. (2013). Impact of atopic dermatitis and loss-of-function mutations in the filaggrin gene on the development of occupational irritant contact dermatitis. Br. J. Dermatol..

[24] Sandilands A., Sutherland C., Irvine A.D., McLean W.I. (2009). Filaggrin in the frontline: Role in skin barrier function and disease. J. Cell Sci..

[25] Kottner J., Surber C. (2016). Skin care in nursing: A critical discussion of nursing practice and research. Int. J. Nurs. Stud..

[26] Ma Q.X., Shan H., Zhang H.L., Li G.M., Yang R.M., Chen J.M. (2020). Potential utilities of mask-wearing and instant hand hygiene for fighting SARS-CoV-2. J. Med. Virol..

[27] Giacalone S., Bortoluzzi P., Nazzaro G. (2020). The fear of COVID-19 infection is the main cause of the new diagnoses of hand eczema: Report from the frontline in Milan. Dermatol. Ther..

[28] Singh M., Pawar M., Bothra A., Choudhary N. (2020). Overzealous hand hygiene during the COVID 19 pandemic causing an increased incidence of hand eczema among general population. J. Am. Acad. Dermatol..

[29] Lee W.J., Kim J.Y., Song C.H., Jung H.D., Lee S.H., LEE S.J., Kim D.W. (2011). Disruption of barrier function in dermatophytosis and pityriasis versicolor. J. Dermatol..

[30] Leggit J.C. (2017). Acute and chronic paronychia. Am. Fam. Physician.

[31] Kutlu Ö., Metin A. (2020). Dermatological diseases presented before COVID-19: Are patients with psoriasis and superficial fungal infections more vulnerable to the COVID-19?. Dermatol. Ther..

[32] Galadari H., Gupta A., Kroumpouzos G., Kassir M., Rudnicka L., Lotti T., Berg R.V., Goldust M. (2020). COVID 19 and its impact on cosmetic dermatology. Dermatol. Ther..

[33] Valenti M., Pavia G., Gargiulo L., Facheris P., Nucca O., Mancini L., Sacrini F., Borroni R.G., Narcisi A., Costanzo A. (2021). Impact of delay in follow-up due to COVID-19 pandemic on skin cancer progression: A real-life experience from an Italian hub hospital. Int. J. Dermatol..

[34] Puavilai S., Noppakun N., Sitakalin C., Leenutaphong V., Wattanakrai P., Nakakes A., Kulthanan K., Asawanonda P., Akaraphan R., Tresukosol P. (2005). Drug eruptions at five institutes in Bangkok. J. Med. Assoc. Thai..

[35] Saengcharoen W., Chongsuvivatwong V., Lerkiatbundit S., Wongpoowarak P. (2008). Factors influencing dispensing of antibiotics for upper respiratory infections among Southern Thai community pharmacists. J. Clin. Pharm. Ther..

[36] Sirijoti K., Hongsranagon P., Havanond P., Pannoi W. (2014). Assessment of knowledge attitudes and practices regarding antibiotic use in Trang province, Thailand. J. Health Res..

[37] Lin C.F., Huang Y.H., Cheng C.Y., Wu K.H., Tang K.S., Chiu I.M. (2020). Public health interventions for the COVID-19 pandemic reduce respiratory tract infection-related visits at pediatric emergency departments in Taiwan. Front. Public Health.

[38] Chaabna K., Doraiswamy S., Mamtani R., Cheema S. (2020). Facemask use in community settings to prevent respiratory infection transmission: A rapid review and meta-analysis. Int. J. Infect. Dis..

